# Decoronation-induced infected alveolar socket defect rat model for ridge preservation

**DOI:** 10.1038/s41598-022-14064-6

**Published:** 2022-06-15

**Authors:** Chih-Hsiang Fang, Hung-Ying Lin, Chung-Kai Sun, Yi-Wen Lin, Min-Chih Hung, Ching-Hung Li, I-Ping Lin, Hung-Chen Chang, Jui-Sheng Sun, Jenny Zwei-Chieng Chang

**Affiliations:** 1 Trauma and Emergency Center, China Medical University Hospital, No. 2, Xueshi Rd., North Dist., Taichung City, 404018 Taiwan; 2 Department of Dentistry, College of Medicine, National Taiwan University Hospital, No. 1, Chang-Te Street, Taipei, 10048 Taiwan; 3 Institute of Traditional Medicine, School of Medicine, National Yang Ming Chiao Tung University, No. 155, Sec. 2, Linong Street, Taipei, 11221 Taiwan; 4 Institute of Biomedical Engineering, College of Medicine and College of Engineering, National Taiwan University, No. 1, Sec. 4, Roosevelt Rd, Taipei, 10617 Taiwan; 5 Department of Dentistry, National Taiwan University Hospital, Hisnchu Branch, No. 25, Lane 442, Sec. 1 Jingguo Rd., Hsinchu City, 30059 Taiwan; 6 Gin Chen Dental Clinic, No. 31, Long Chiang Rd, Taipei, Taiwan; 7 School of Medicine, China Medical University-YingCai Campus, No. 91, Xueshi Rd., North Dist., Taichung City, 404333 Taiwan; 8 Department of Orthopedic Surgery, National Taiwan University Hospital, No. 7, Chung-Shan South Road, Taipei, 10002 Taiwan; 9 School of Dentistry, College of Medicine, National Taiwan University, No 1, Chang-Te Street, Taipei, 10048 Taiwan; 10 Department of Orthopedic Surgery, College of Medicine, China Medical University, No. 2, Yu-Der Rd, Taichung City, 40447 Taiwan

**Keywords:** Health care, Medical research

## Abstract

Current rat alveolar ridge preservation models have not been well standardized. In this study, we proposed decoronation-induced infected alveolar socket model of rat. The bilateral maxillary first molars (M1) of twenty-four rats were decoronized or extracted. After 2, 6, 10, and 14 weeks, bone and soft tissue changes at M1 and periodontal conditions of maxillary second (M2) and third molars (M3) were evaluated by micro-computed tomography and histological analysis. Additional eighteen rats with standardized size defects were grafted with Bio-Oss Collagen to compare with unmanipulated contralateral side. Decoronation preserved greater bone and soft tissue dimensions at M1, provided larger three-dimensional (3D) bone contour volume, but also promoted periodontal breakdown of M2 Histological results showed intense inflammatory cell infiltrations and severe bone resorption within M1 socket and at mesial aspect of M2. The critical dimensions to accommodate largest standardized defect at M1 were 2.2–2.3 mm at vertical bone height and 2.8–3.2 mm at alveolar crestal width. Bio-Oss Collagen could not fully preserve buccal or palatal bone height but could be beneficial in preserving ridge width in large alveolar defects. Collectively, if periodontally-involved alveolar bone defect is preferred, we suggest extracting M1 roots 6 weeks after decoronation to allow periodontitis to occur at M2. If standardized critical dimension defect is preferred, we suggest extracting M1 roots 2 weeks after decoronation, and creating defect in the middle of M1 site with size no larger than 2.7 mm diameter to its full depth.

## Introduction

Alveolar ridge undergoes resorption concomitantly with shrinkage of soft tissues after tooth loss^1^. The reduction in alveolar ridge dimension often interferes with subsequent prosthodontic treatment and endosseous dental implant therapy. Results from previous studies have concluded that, during post-extraction healing period, the clinical loss in alveolar ridge width is greater than the loss in height^2–5^. These results demonstrate that alveolar bone resorption after tooth loss occurs in all dimensions. To attenuate the resorptive rate of alveolar ridge and to minimize ancillary ridge augmentation procedures, preservation of the alveolus at the time of extraction has been proposed. Results from meta-analyses show that alveolar ridge preservation (ARP) may preserve bucco-oral bone width and bone height in comparison with unassisted socket healing^6^. At present, no recommendation can be made referring to which ARP technique or biomaterial is the best^6,7^, there is still a need for research to improve the current ARP protocols and materials.

Preclinical testing of any new ARP therapy in clinically relevant animal model is indispensable. To evaluate bone substitute biomaterials or bone regenerative modalities in a standardized and discriminative manner, the critical size defect (CSD) has been widely used as ‘proof-of-principle’ model^8^. Several critical size defect rat models have been developed in the craniomandibulofacial bones, including the calvaria, mandible, and maxilla^9–11^, most of these defects were in fact not created in the tooth-bearing alveolar bone and reflect poorly on the clinical scenario of alveolar bone defects. Regarding the defect dimensions, most CSD studies only reported the diameter but not the depth of the defects. To evaluate the reconstructive modalities that aim to enhance bone regeneration and preserve alveolar dimension following tooth extraction, alveolar socket (tooth extraction) model should be established instead.

In rat models, tooth extractions are usually performed on incisors or maxillary molars without further enlargement of the sockets. The small dimension of the extraction socket and strong innate healing capacity make this particular extraction model less suitable for screening new medical formulations^12^. The dimensions of the socket defects are not standardized and far from ‘critical size’. To evaluate various alveolar ridge preservation/regeneration modalities in rats, some researchers enlarged the socket to a standardized size following extraction^13–16^. Taken together, alveolar ridge preservation models in rats have not been well standardized in the literature. From a clinical perspective, it would be more suitable to use infected defect models^17,18^. The occurrence of periodontal diseases in rats is less frequent than in human. To induce the periodontal pathology, rats often have to receive inoculation of bacteria, a carbohydrate-rich diet, and ligatures fixed around the teeth^19^.

Since rat alveolar ridge preservation models have not yet been standardized and mostly not clinically-relevant, we presented a decoronation-induced infected alveolar socket model of rat, which further divided into the periodontally-involved alveolar socket defect and the standardized critical dimension socket defect models. This current proof-of-principle study proposed a simple and cost-effective way to develop infected alveolar socket defect in rats. The technique involves decoronation of the maxillary first molar (M1). The residual M1 roots have induced inflammation in the socket. Complete and atraumatic removal of the five roots may be easily preformed later. In comparison with the contralateral extraction side (a split-mouth design), the decoronation process has preserved the bone contour volume of M1 despite the severe bone destruction in the infected socket. The soft tissue height was also preserved. The greater hard and soft tissue dimensions allowed accommodation of increased amount of grafting materials and tension-free primary closure if future bone grafting procedures are planned. The proximity of the residual distal roots of M1 with the mesial roots of second molar (M2) has accelerated the development of periodontitis at M2. We further provided critical alveolar defect dimensions to accommodate maximum amount of grafting material if standardized size defect is preferred. Additional rats have been used as examples to evaluate the effect of ridge preservation using Bio-Oss Collagen in this large critical dimension alveolar socket defect model.

## Materials and methods

### In vivo* animal study*

All procedures were pre-approved and supervised under the Institutional Animal Care and Use Committee (IACUC) of the National Taiwan University College of Medicine and College of Public Health (Affidavit of Approval of Animal Use Protocol: 20,190,084). All the procedures were performed in accordance with the ARRIVE guidelines. The in vivo animal study was further separated into two parts: the periodontally-involved infected alveolar socket defect model and the standardized critical dimension socket defect model.

### Part I: infected alveolar socket defect model

A total of 24 8-week-old Sprague–Dawley (SD) male rats, weighing 200–250 g, were anesthetized with a 1:2 concentration of Zoletil (Virbac AH, Inc., Fort Worth, TX, USA) and Rompum (Bayer, Taipei, Taiwan) (1 mL/kg) via intraperitoneal injection. Local anesthesia (Articaine 40 mg, 1% epinephrine; Merck KGaA, Darmstadt, Germany) was performed at each intraoral site for additional pain control and blood hemostasis. On one side, the mucosa was aseptically prepared, the maxillary M1 was decoronized to the furcation level to separate the five roots, and the soft tissue was to heal by secondary healing. On the contralateral side, the maxillary M1 was extracted entirely using a scaler (split-mouth design) on the same day. A total of 48 M1 teeth were decoronized or extracted from 24 rats and euthanized at 2, 6, 10, and 14 weeks.

### Part II: standardized critical dimension socket defect model

Standardized size alveolar bone defects were created in additional 18 eight-week-old SD rats 2 weeks after decoronation and grafted with Bio-Oss Collagen (90% Spongious Bio-Oss bone substitute granules + 10% porcine collagen; Geistlich Biomaterials, Wolhusen, Switzerland) on one side to compare with the unmanipulated (non-extraction) contralateral side. In brief, decoronation of the maxillary M1 was first performed till separation of the roots. After 2 weeks of secondary soft tissue healing, a semilunar incision was made to expose the alveolar crest and the remaining M1 roots were completely removed. A standardized alveolar bone defect was created using low-speed tungsten carbide round bur (ISO 027, diameter 2.7 mm) with low-speed dental handpiece (1000 rpm; SM, Osstem, Taiwan) under continuous saline irrigation by aiming the center of inter-radicular septa till the depth that the bur head just submerged into the level of soft tissue surface (i.e. 2.7 mm). Primary closure was achieved using resorbable vicryl 6–0 sutures after grafting the bone defects with Bio-Oss Collagen. The rats were euthanized at 4, 8, and 12 weeks after grafting (six rats per time-point). Throughout the entire observation period, the animals were monitored once per day for clinical complications or side effects and soft diet was prescribed.

### Micro-computed tomography (micro-CT) assessments

**Figure 1:**
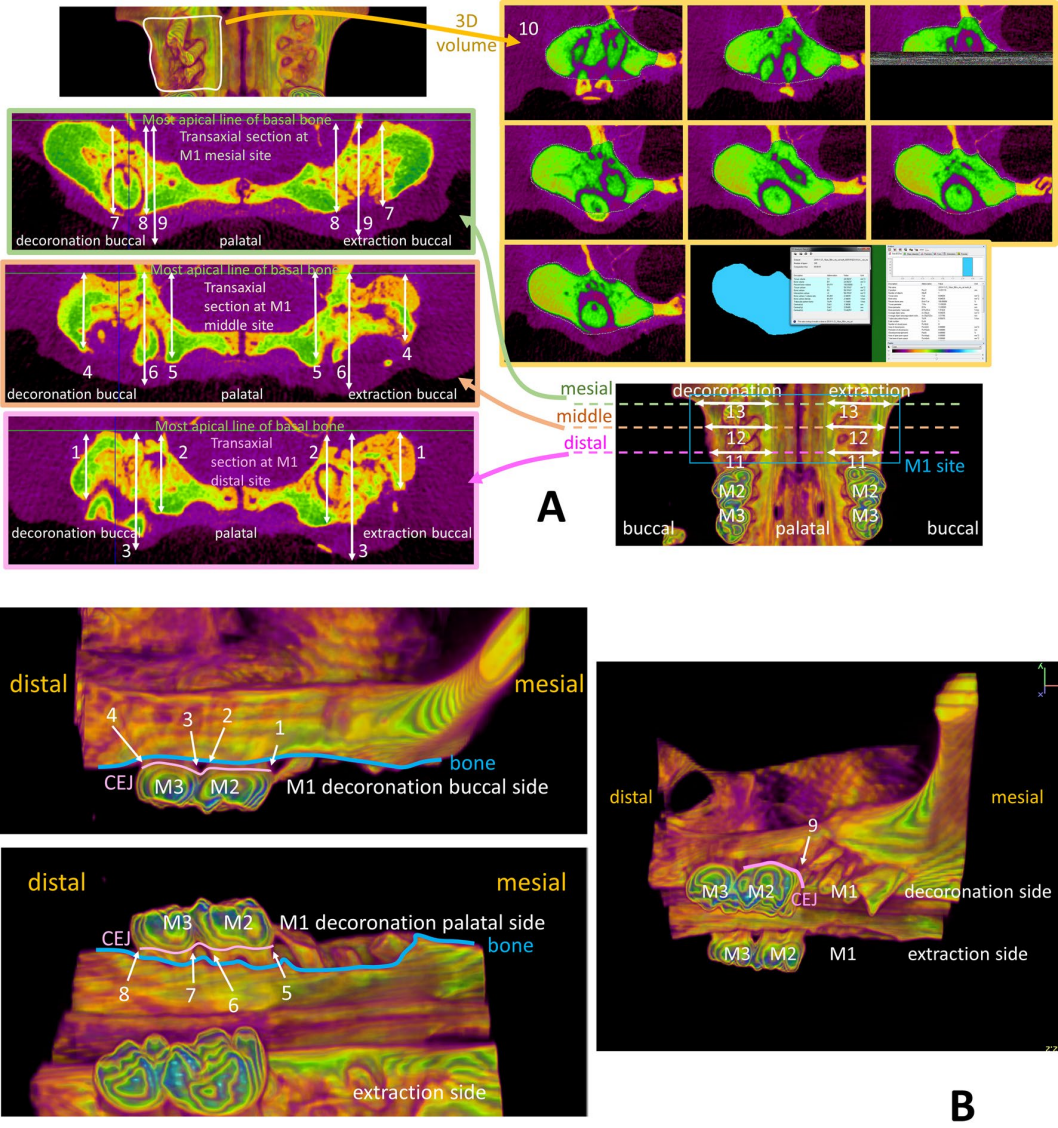

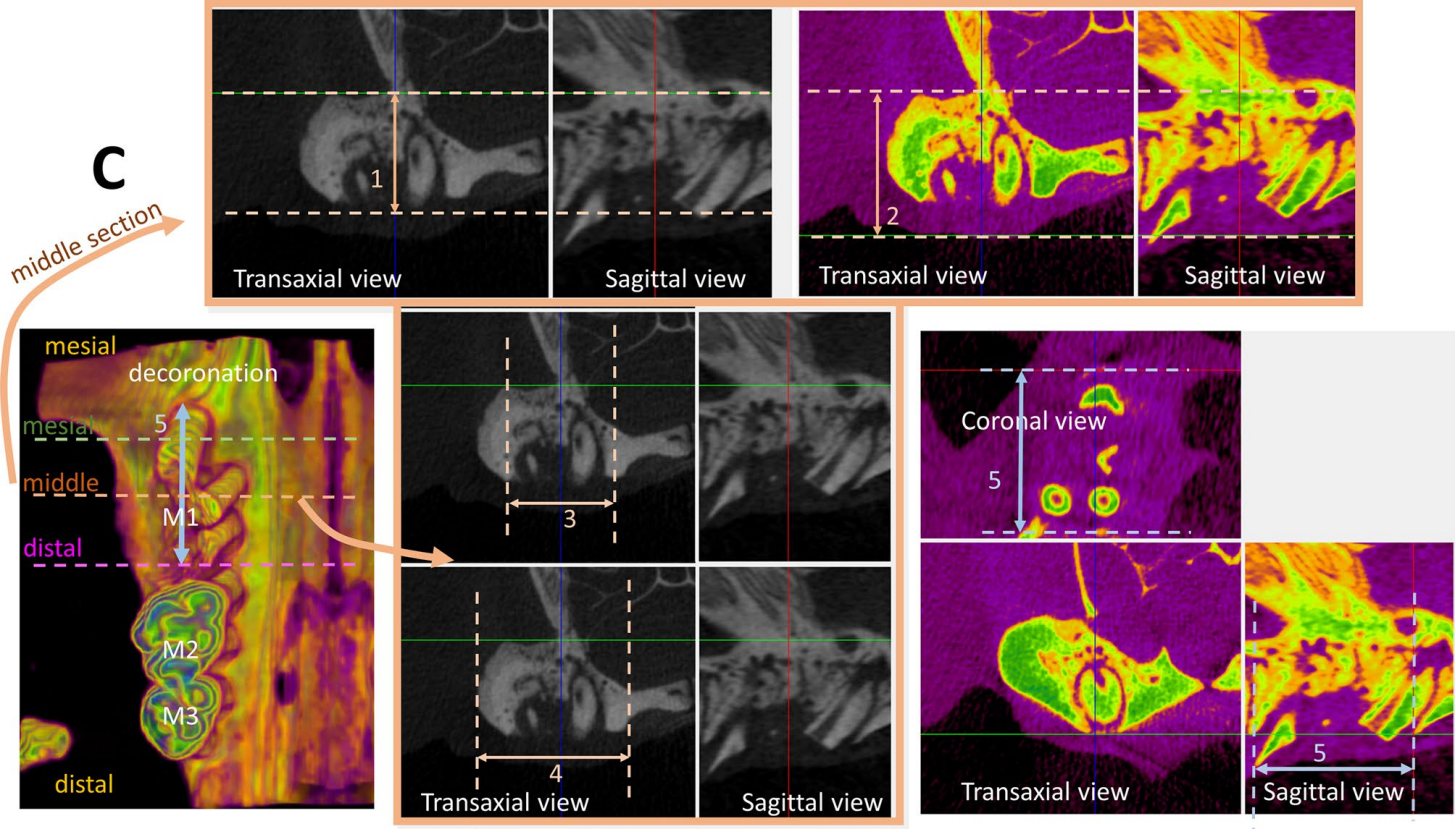
Micro-computed tomography assessments of alveolar bone and soft tissue dimension. (**A**) To evaluate the effectiveness of hard and soft tissue preservation of alveolar ridge dimension associated with the decoronation procedures, thirteen parameters were measured at maxillary first molar (M1) sites including (1) distal buccal bone height, (2) distal palatal bone height, (3) distal maximum soft tissue height, (4) middle buccal bone height, (5) middle palatal bone height, (6) middle maximum soft tissue height, (7) mesial buccal bone height, (8) mesial palatal bone height, (9) mesial maximum soft tissue height, (10) 3D bone contour volume*, (11) distal maximum alveolar ridge width, (12) middle maximum alveolar ridge width, and (13) mesial maximum alveolar ridge width. (**B**) To evaluate the influence of decoronation on the periodontal conditions of maxillary second molar (M2) and third molar (M3), the distances from marginal bone to cemento-enamel junction (CEJ) including (1) bM2mCEJ: M2 mesiobuccal CEJ to bone; (2) bM2dCEJ: M2 distobuccal CEJ to bone; (3) bM3mCEJ: M3 mesiobuccal CEJ to bone; (4) bM3dCEJ: M3 distobuccal CEJ to bone; (5) pM2mCEJ: M2 mesiopalatal CEJ to bone; (6) pM2dCEJ: M2 distopalatal CEJ to bone; (7) pM3mCEJ: M3 mesiopalatal CEJ to bone; (8) pM3dCEJ: M3 distopalatal CEJ to bone; and (9) the distance from CEJ to mesial furcation of M2 (M2mf) were measured. (**C**) To provide reference for alveolar critical size defect dimensions in a standardized and discriminative manner, five parameters were measured at the decoronated M1 site, including (1) middle bone height, (2) middle tissue height, (3) middle alveolar crestal width, (4) middle maximum bone width, and (5) mesio-distal socket length. *Three-dimensional (3D) bone contour volume was calculated on either side by adding up consecutive surfaces of interest on the transaxial section mesio-distally throughout the M1 site.

The rats were anesthetized and then sacrificed at designated time-points by intracardiac injection of 150 mg/kg potassium chloride (Taiwan Biotec Co., Ltd., Taoyuan, Taiwan). The animals were then quantitatively evaluated by high-resolution micro-computed tomography (micro-CT) (Skyscan1076, Kontich, Belgium). To evaluate the effectiveness of hard and soft tissue preservation of alveolar ridge dimension associated with the decoronation procedures, thirteen parameters were measured on the decoronated and extracted maxillary M1 sites (Fig. 1A). To evaluate the influence of decoronation on the periodontal conditions of maxillary M2 and third molar (M3), the distances from marginal bone to cemento-enamel junction (CEJ) at buccal (b), palatal (p), mesial (m) and distal (d) aspects of M2 and M3 teeth, nine parameters (bM2mCEJ: M2 mesiobuccal CEJ to bone, bM2dCEJ: M2 distobuccal CEJ to bone, bM3mCEJ: M3 mesiobuccal CEJ to bone, bM3dCEJ: M3 distobuccal CEJ to bone, pM2mCEJ: M2 mesiopalatal CEJ to bone, pM2dCEJ: M2 distopalatal CEJ to bone, pM3mCEJ: M3 mesiopalatal CEJ to bone, pM3dCEJ: M3 distopalatal CEJ to bone and M2mf: the distance from CEJ to mesial furcation of M2) were measured (Fig. 1B). To provide reference for the critical dimensions of alveolar defect in a standardized and discriminative manner for future research in bone substitute biomaterials or bone regenerative modalities, five additional parameters (middle bone height, middle tissue height, middle alveolar crestal width, middle maximum bone width, and mesio-distal socket length of alveolar ridge dimension) were measured at the maxillary first molar (M1) site (Fig. 1C). A region of interest around each measurement was accurately defined and positioned for quantitative analysis. Then, the quantifications and 3 dimensional (3D) analyses of the alveolar bone from the reconstructed ROI images were performed by DataViewer or CTAn Skyscan software.

### Histological analysis

The specimens were fixed and decalcified by 20% EDTA (adjusted to 7.4 with NaOH; Sigma-Aldrich, USA) for 2 weeks. The specimens were embedded in paraffin, sectioned at a thickness of 5-μm, mounted on glass slide, stained with hematoxylin and eosin (H&E) or Goldner's Trichrome staining, and then examined under a light microscope (IX71; Olympus, Tokyo, Japan) as previously described^20^.

### Statistical analysis

All data were expressed as mean ± standard deviation (SD). Statistical analysis was performed using one-way ANOVA followed by post hoc comparisons with Bonferroni adjustment. Statistically significance was defined at *P*-value less than 0.05. All analyses were performed using SPSS version 16.0 software (SPSS Inc., Chicago, USA).

### Ethics approval and consent to participate

All animal procedures and experiments were performed following a protocol approved by the National Taiwan University College of Medicine and Public Health Institutional Animal Care and Use Committee (IACUC no. 20190084).

### A statement of the location where the work was performed

All works were performed at National Taiwan University, National Taiwan University Hospital (Taipei City, Taiwan).

## Results

All animals survived to the end of the study.

### Micro-CT findings

**Figure 2:**
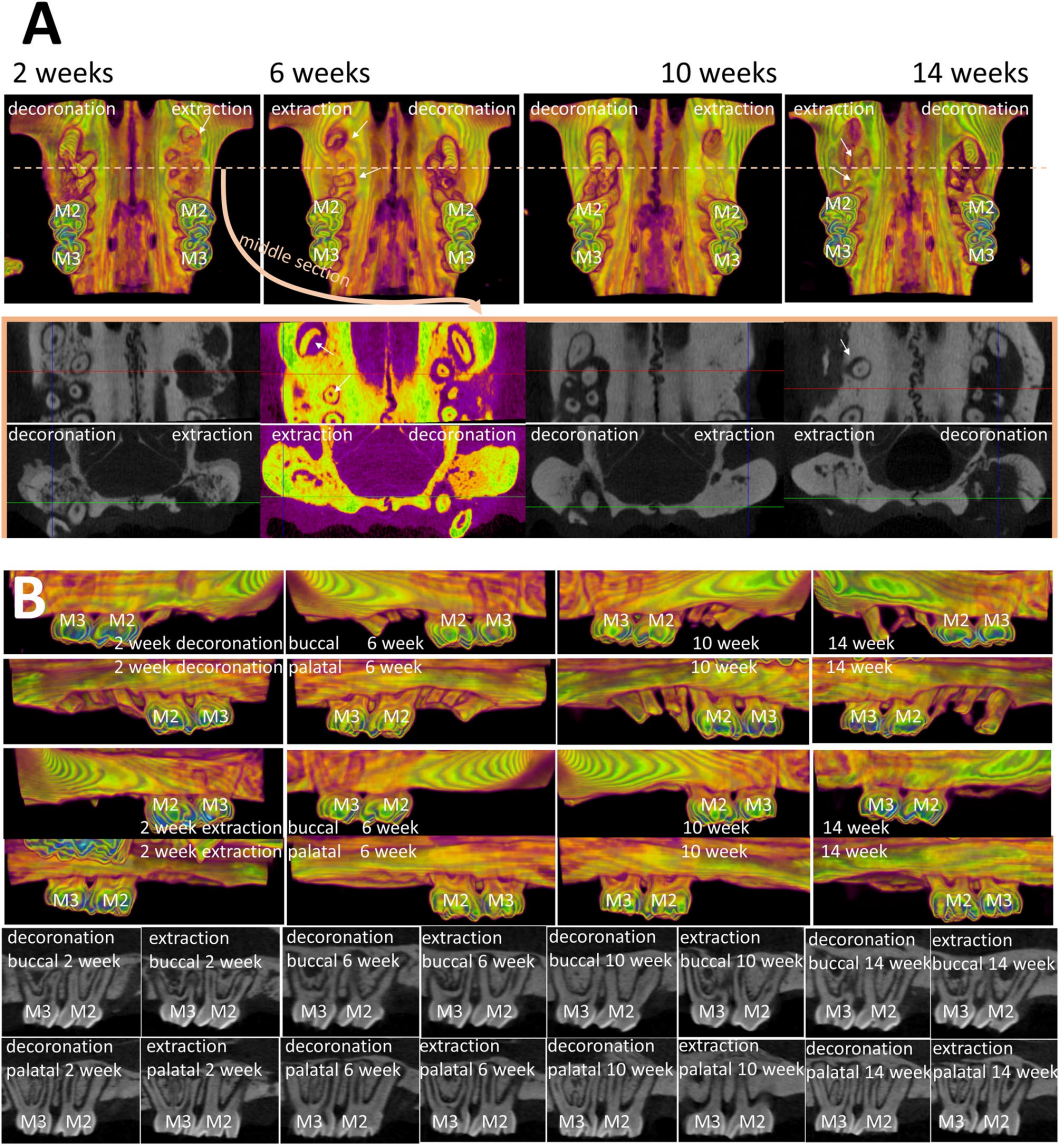
Comparison of the effectiveness of alveolar ridge preservation at maxillary first molar (M1) and the periodontal condition at maxillary second molar (M2) and third molar (M3) after decoronation or extraction of M1. (**A**) Comparison of the effectiveness of alveolar ridge preservation with decoronation technique at maxillary first molar (M1) examined by micro-CT. Representative images show the 3D occlusal view (top), the coronal view, and the transaxial section in the middle of M1 site (bottom) at 2, 6, 10, 14 weeks. Fuller bone contour and better bone height were obvious on the decoronation side compared to the contralateral extraction side. Please note that some root fragments remained in the extraction side (white arrow). (**B**) Comparison of the periodontal condition at maxillary second molar (M2) and third molar (M3) after decoronation or extraction of M1. Representative images show the 3D buccal and palatal views on the decoronation and extraction sides (color images) and 2D sections cut along a profile line through the buccal aspect or palatal aspect of M2 and M3 (black/white images). On the decoronation side, significant more bone resorption may be seen at both buccal and palatal aspects of the M2 teeth, especially at mesial side.

The effectiveness of alveolar bone and soft tissue preservation with decoronation technique was examined by micro-CT at 2, 6, 10, 14 weeks comparing the decoronation and contralateral extraction M1 sites (Table 1). At the distal site, there was not much difference between the decoronation and extraction sides, except that the decoronation side appeared to have a wider ridge at 2 week. At the middle site, the decoronation side exhibited substantially greater amount of buccal bone height at 6th and 10th week and soft tissue height at 6th, 10th, and 14th week (Fig. 2A). At the mesial site, the decoronation side showed greater buccal and palatal bone heights and soft tissue height at almost all time-points. Three-dimensional bone contour volume was calculated on either side by adding up consecutive surfaces of interest on the transaxial section mesio-distally throughout the M1 site. The results revealed considerably larger 3D volume at the decoronation side at all time-points, indicating preservation of alveolar ridge dimension with the decoronation technique.

**Table 1:** Micro-computed tomography assessments of alveolar bone and soft tissue dimension comparing the decoronation (Decor.) and contralateral extraction (Ext.) maxillary first molar sites at 2, 6, 10, and 14 weeks.

	2 week			6 week			10 week			14 week		
	Decor.	Ext.	*P*-value	Decor.	Ext.	*P*-value	Decor.	Ext.	*P*-value	Decor.	Ext.	*P*-value
Distal buccal bone height (mm)	1.99 ± 0.19	1.75 ± 0.14	0.035*	2.09 ± 0.15	1.71 ± 0.14	0.015*	2.11 ± 0.13	1.89 ± 0.11	0.014*	2.11 ± 0.18	1.86 ± 0.17	0.014*
Distal palatal bone height (mm)	2.44 ± 0.19	2.36 ± 0.11	0.182	2.49 ± 0.20	2.38 ± 0.12	0.313	2.56 ± 0.28	2.44 ± 0.18	0.077	2.53 ± 0.12	2.39 ± 0.07	0.161
Distal max soft tissue height (mm)	3.55 ± 0.29	3.33 ± 0.15	0.142	3.67 ± 0.22	3.34 ± 0.10	0.064	3.72 ± 0.22	3.44 ± 0.22	0.104	3.72 ± 0.42	3.37 ± 0.15	0.341
Middle buccal bone height (mm)	2.27 ± 0.16	2.07 ± 0.27	0.058	2.33 ± 0.21	2.10 ± 0.11	0.031*	2.47 ± 0.18	2.20 ± 0.25	0.018*	2.35 ± 0.15	2.07 ± 0.21	0.031*
Middle palatal bone height (mm)	2.69 ± 0.24	2.56 ± 0.19	0.234	2.72 ± 0.21	2.58 ± 0.22	0.262	2.81 ± 0.24	2.69 ± 0.23	0.450	2.81 ± 0.24	2.60 ± 0.17	0.172
Middle max soft tissue height (mm)	3.69 ± 0.18	3.44 ± 0.12	0.090	3.70 ± 0.28	3.53 ± 0.29	0.074	4.05 ± 0.19	3.67 ± 0.25	0.087	3.91 ± 0.36	3.58 ± 0.29	0.015*
Mesial buccal bone height (mm)	2.94 ± 0.47	2.49 ± 0.14	0.010*	3.06 ± 0.29	2.56 ± 0.32	0.014*	3.19 ± 0.25	2.75 ± 0.09	0.018*	3.09 ± 0.44	2.65 ± 0.54	0.014*
Mesial palatal bone height (mm)	3.09 ± 0.29	2.64 ± 0.14	0.013*	3.23 ± 0.32	2.92 ± 0.16	0.052	3.31 ± 0.28	3.05 ± 0.23	0.079	3.30 ± 0.23	2.98 ± 0.27	0.09
Mesial max soft tissue height (mm)	4.09 ± 0.38	3.58 ± 0.16	0.042*	4.22 ± 0.34	3.82 ± 0.36	0.053	4.26 ± 0.29	3.98 ± 0.36	0.06	4.25 ± 0.40	3.92 ± 0.16	0.08
3D bone contour volume (mm^3^)	23.42 ± 3.59	19.14 ± 1.13	0.018*	24.33 ± 0.86	20.11 ± 2.77	0.017*	28.81 ± 3.19	22.37 ± 1.15	0.007*	25.10 ± 3.58	21.83 ± 3.20	0.013*
Distal max alveolar ridge width (mm)	3.85 ± 0.25	3.58 ± 0.25	0.078	3.90 ± 0.35	3.62 ± 0.18	0.135	4.05 ± 0.33	3.84 ± 0.23	0.148	3.95 ± 0.32	3.80 ± 0.26	0.313
Middle max alveolar ridge width (mm)	4.18 ± 0.36	3.83 ± 0.14	0.110	4.20 ± 0.23	3.86 ± 0.08	0.114	4.42 ± 0.43	4.16 ± 0.29	0.356	4.34 ± 0.34	3.94 ± 0.17	0.210
Mesial max alveolar ridge width (mm)	4.34 ± 0.16	3.77 ± 0.29	0.014*	4.32 ± 0.25	3.95 ± 0.16	0.320	4.36 ± 0.41	4.15 ± 0.21	0.406	4.40 ± 0.54	4.14 ± 0.40	0.457

n = 6 per group; Statistically significantly different at *P < 0.05; Data are represented as mean ± standard deviation.

To quantitate the amount of bone loss associated with decoronation versus extraction, the distances from CEJ to marginal bone crest were measured on the mesial/distal and buccal/palatal aspects of M2 and M3 and at the mesial furcation area of M2. Results showed significant more bone loss in the mesio-buccal (10th week), mesio-palatal (6th, 10th, 14th week), mesial furcation (2nd, 10th, 14th week), disto-buccal (10th week), and disto-palatal (10th, 14th week) aspects of M2 on the decoronation side compared to the extraction side (Table 2 and Fig. 2B). Significant bone loss was also found at M3 on the decoronation side in the mesio-buccal (2nd, 10th week) and mesio-palatal (10th, 14th week) aspects. However, the results at M3 distal sites were not that consistent, as the coronation side showed more bone loss in the disto-buccal at 10th week and disto-palatal at 2nd week, while the extraction side showed more bone loss in the disto-buccal at 14th week) and disto-palatal at 6th week.

**Table 2:** Micro-computed tomography assessments of the distances from marginal bone to cemento-enamel junction (CEJ) at buccal (b), palatal (p), mesial (m), and distal (d) aspects of maxillary second molar (M2) and third molar (M3) and the distance from CEJ to mesial furcation of M2 (M2mf) comparing the decoronation (Decor.) and contralateral extraction (Ext.) sides at 2, 6, 10, and 14 weeks.

	2 week			6 week			10 week			14 week		
	Decor.	Ext.	*P*-value	Decor.	Ext.	*P*-value	Decor.	Ext.	*P*-value	Decor.	Ext.	*P*-value
bM2mCEJ (mm)	0.47 ± 0.07	0.38 ± 0.04	0.014*	0.82 ± 0.10	0.52 ± 0.07	0.000*	1.05 ± 0.13	0.69 ± 0.08	0.000*	0.93 ± 0.13	0.64 ± 0.08	0.001*
bM2dCEJ (mm)	0.39 ± 0.08	0.28 ± 0.05	0.001*	0.42 ± 0.07	0.31 ± 0.05	0.007*	0.43 ± 0.07	0.32 ± 0.05	0.005*	0.42 ± 0.05	0.31 ± 0.04	0.008*
bM3mCEJ (mm)	0.36 ± 0.05	0.18 ± 0.03	0.000*	0.36 ± 0.05	0.25 ± 0.04	0.000*	0.38 ± 0.06	0.28 ± 0.04	0.000*	0.36 ± 0.05	0.26 ± 0.04	0.003*
bM3dCEJ (mm)	0.24 ± 0.03	0.17 ± 0.03	0.006*	0.26 ± 0.03	0.18 ± 0.03	0.000*	0.30 ± 0.04	0.20 ± 0.03	0.000*	0.28 ± 0.05	0.19 ± 0.02	0.000*
pM2mCEJ (mm)	0.68 ± 0.07	0.47 ± 0.07	0.001*	1.04 ± 0.11	0.46 ± 0.09	0.000*	1.16 ± 0.13	0.64 ± 0.09	0.000*	1.25 ± 0.12	0.63 ± 0.08	0.000*
pM2dCEJ (mm)	0.46 ± 0.07	0.34 ± 0.04	0.003*	0.51 ± 0.05	0.35 ± 0.04	0.000*	0.59 ± 0.07	0.44 ± 0.05	0.003*	0.56 ± 0.08	0.44 ± 0.06	0.002*
pM3mCEJ (mm)	0.36 ± 0.05	0.22 ± 0.03	0.007*	0.39 ± 0.05	0.23 ± 0.03	0.000*	0.44 ± 0.05	0.35 ± 0.04	0.007*	0.43 ± 0.08	0.33 ± 0.04	0.003*
pM3dCEJ (mm)	0.26 ± 0.03	0.20 ± 0.03	0.003*	0.26 ± 0.04	0.24 ± 0.04	0.026*	0.32 ± 0.04	0.25 ± 0.03	0.017*	0.29 ± 0.04	0.25 ± 0.03	0.013*
M2mf (mm)	0.45 ± 0.07	0.37 ± 0.05	0.002*	0.47 ± 0.06	0.38 ± 0.04	0.019*	0.57 ± 0.07	0.43 ± 0.06	0.003*	0.50 ± 0.05	0.40 ± 0.05	0.018*

n = 6 per group; Statistically significantly different at *P < 0.05; Data are represented as mean ± standard deviation.

To determine the largest alveolar defect that may be created to accommodate maximum amount of bone substitute biomaterials, five additional parameters (middle bone height, middle tissue height, middle alveolar crestal width, middle maximum bone width, and mesio-distal socket length) were measured at the M1 site. The measurements on middle bone height, middle soft issue height, and middle alveolar crestal width of the decoronated side were significantly larger than those of extracted side and mostly maintained throughout different time-points; while the measurements of the extracted side showed gradual decrease with time. There was not much difference between the middle maximum bone width (measured at the level of basal bone) and mesio-distal socket length on both sides (Table 3). Although decoronation has preserved ridge dimension and provided a bigger housing to accommodate various grafts for future research reprojects, the residual roots should be extracted subsequently and standardized size defect may then be created. Therefore, the mean ± SD of the following measurements were provided on the decoronated M1 sites at different time-points. As shown in Table 3, middle bone height ranged from 2.156 ± 0.262 mm (6th week) to 2.319 ± 0.174 mm (14th week); middle soft tissue height ranged from 3.094 ± 0.077 mm (10th week) to 3.429 ± 0.343 mm (14th week); middle alveolar crestal width ranged from 2.808 ± 0.075 mm (10th week) to 3.180 ± 0.333 mm (2nd week); middle maximum bone width ranged from 3.533 ± 0.228 mm (10th week) to 4.221 ± 0.107 mm (14th week); and mesio-distal socket length ranged from 3.088 ± 0.134 mm (6th week) to 3.600 ± 0.201 mm (2nd week).

**Table 3:** Micro-computed tomography measurements of alveolar ridge dimension at the maxillary first molar site 2, 6, 10, or 14 weeks after decoronation (Decor.) or extraction (Ext.).

	2 week			6 week			10 week			14 week		
	Decor.	Ext.	*P*-value	Decor.	Ext.	*P*-value	Decor.	Ext.	*P*-value	Decor.	Ext.	*P*-value
Middle bone height (mm)	2.21 ± 0.21	2.03 ± 0.10	0.020*	2.16 ± 0.29	1.93 ± 0.16	0.012*	2.22 ± 0.11	1.69 ± 0.16	0.000*	2.31 ± 0.19	1.85 ± 0.237	0.005*
Middle tissue height (mm)	3.16 ± 0.18	2.92 ± 0.12	0.001*	3.13 ± 0.178	3.03 ± 0.21	0.026*	3.09 ± 0.08	2.75 ± 0.12	0.007*	3.43 ± 0.38	3.07 ± 0.34	0.001*
Middle alveolar crestal width (mm)	3.18 ± 0.37	2.41 ± 0.16	0.008*	2.91 ± 0.14	2.44 ± 0.35	0.009*	2.81 ± 0.08	2.24 ± 0.27	0.001*	3.10 ± 0.28	2.35 ± 0.36	0.000*
Middle maximum bone width (mm)	3.84 ± 0.21	3.68 ± 0.15	0.039*	4.00 ± 0.55	3.95 ± 0.48	0.116	3.87 ± 0.28	3.62 ± 0.14	0.2	4.22 ± 0.12	4.14 ± 0.42	0.649
Mesio-distal socket length (mm)	3.60 ± 0.22	3.50 ± 0.27	0.005*	3.09 ± 0.15	3.06 ± 0.12	0.175	3.16 ± 0.03	3.11 ± 0.07	0.054	3.18 ± 0.19	3.09 ± 0.06	0.325

Statistically significant at * P< 0.05.

n = 6 per group; Data are represented as mean ± standard deviation.

### Histological findings

**Figure 3:**
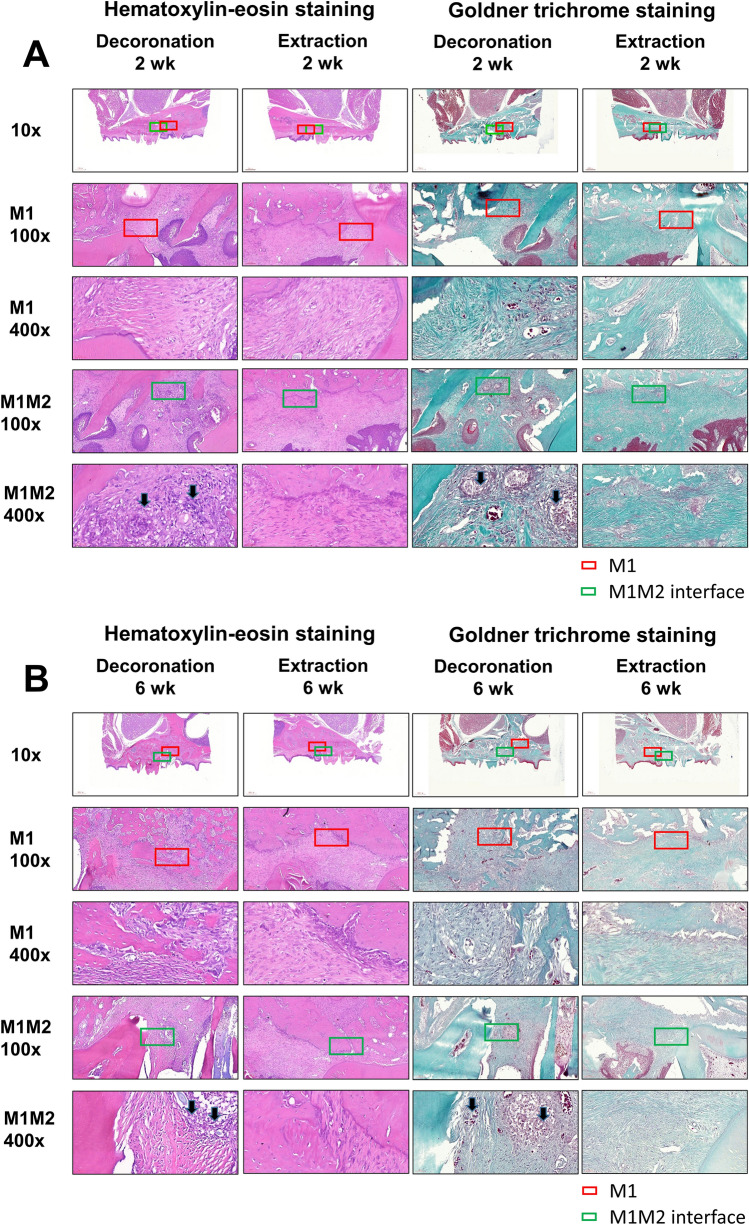

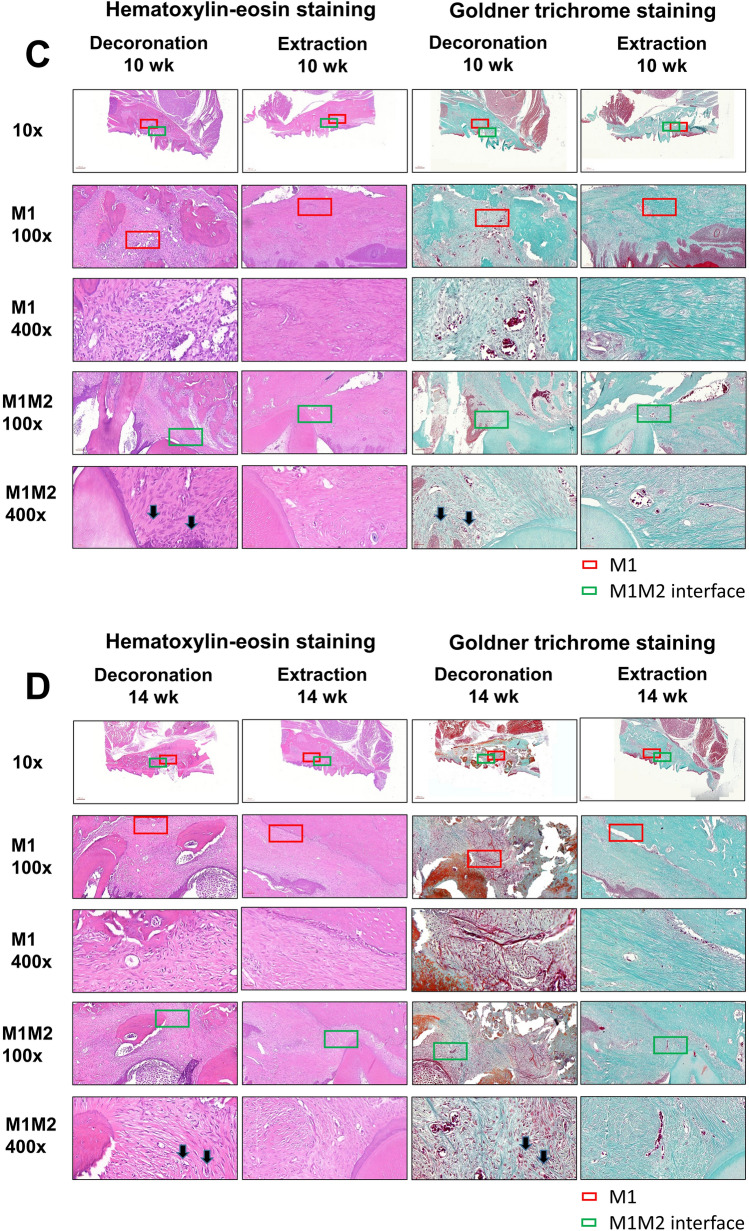
Representative histological sections of the maxillary first molar (M1) to third molar (M3) region of rats after after M1 decoronation or extraction; hematoxylin and eosin (H&E) or Goldner's Trichrome staining. Sections were identified and photographed under 10x, 100x, and 400 × magnifications. Six weeks after M1 extraction, there were still some inflammatory cell infiltrations in the samples; however, these infiltrates decreased a lot at 10th and 14th weeks after extraction. The decoronation samples were dominated by inflammatory granulation tissues, blood vessels with different calibers, and inflammatory cell infiltrations at 6–14 weeks; moreover, intensive alveolar bone resorption activity was characterized by the presence of a border of osteoclasts (arrow) at the M1-M2 junction (green box). With Goldner's Trichrome staining, the collagenous fiber, fibrin and erythrocytes were selectively visualized. The fibrous connective tissue with the presence of granulation tissue with excessive inflammatory cell infiltrations were clearly demonstrated in the decoronation samples.

The histopathological changes after M1 decoronation or extraction were identified by hematoxylin and eosin (H&E) or Goldner’s Trichrome staining. In the extraction sites, there were some inflammatory cell infiltration at 6th week; but the infiltrates decreased greatly at 10th and 14th weeks while fibrous connective tissue formation was also observed. However, inflammatory granulation tissues, blood vessels with different calibers, and inflammatory cell infiltration dominated at 6th, 10th, and 14th weeks at the decoronation sites (M1 alveolar socket area; red box). Severe alveolar bone resorption was also detected and characterized by the presence of a border of osteoclasts (red box) mesial to M2 (M1–M2 junction; green box (Fig. 3). In the Goldner's Trichrome staining, the collagenous fiber, fibrin and erythrocytes were selectively visualized. The fibrous connective tissue with the presence of granulation tissue and intense inflammatory cell infiltrations were clearly demonstrated in the decoronation samples (Fig. 3).

**Figure 4:**
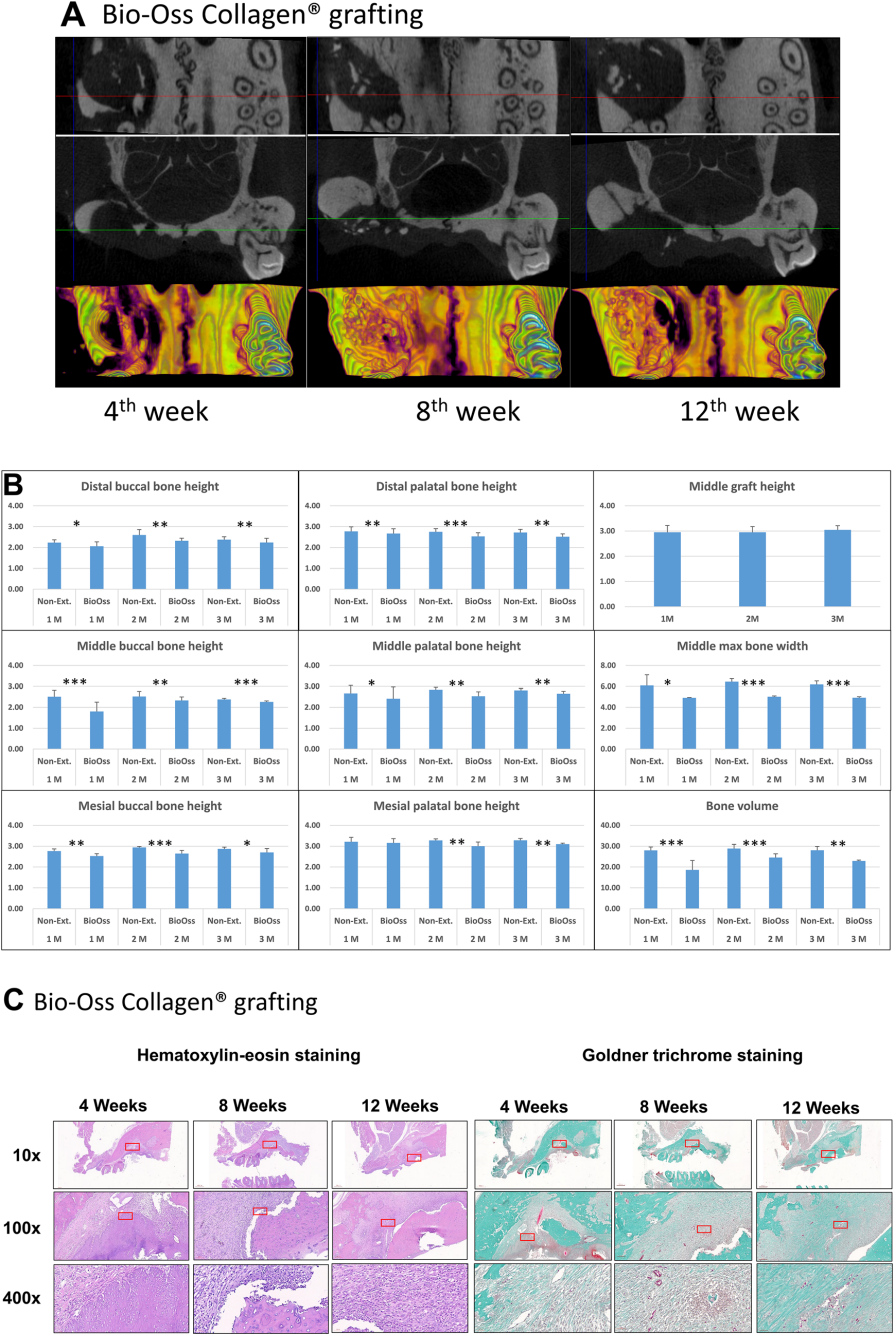
Micro-computed tomography (micro-CT) and histo-morphological evaluation of alveolar ridge bone preservation in standardized critical dimension socket defect model. The effectiveness of alveolar ridge bone preservation at maxillary first molar (M1) examined by micro-computed tomography (micro-CT) after creating large alveolar defect 2 weeks after decoronation and grafted with Bio-Oss Collagen. (**A**) Representative images show the coronal (top) and transaxial (middle) sections in the middle part of M1 site and the 3D occlusal view (bottom) after Bio-Oss Collagen grafting for 4th, 8th, and 12th weeks. Please note that residual grafting material at the defect site was still evident after 12 weeks. (**B**) Analyses of bone heights, middle maximum bone width, and bone volume at different time points. Graft heights were measured in the middle section of M1 site on the grafted side only. Bone volume was measured on the grafted side by adding up consecutive bone surfaces excluding the graft material on the transaxial sections mesio-distally throughout the M1 site. (**C**) Representative histological sections of the maxillary first molar (M1) to third molar (M3) region of rats after M1 decoronation, roots extraction and grafting with Bio-Oss Collagen by hematoxylin & eosin (H&E) and Goldner's Trichrome staining at 4th, 8th, and 12th weeks. At 12th week after grafting, the defects grafted with Bio-Oss Collagen showed some bone regeneration in the defect site; however, the rate of new bone regeneration or mineralization of new bone were still not satisfactory at 12th week. Goldner's trichrome staining shows immature bone formation and soft tissue invasion were observed but no angiogenesis found.

The effectiveness of alveolar ridge preservation with Bio-Oss Collagen was examined by micro-CT comparing the grafting and contralateral non-extraction M1 sites. Representative coronal and tranaxial sections and 3D occlusal view of M1 grafting sites at 4th, 8th, and 12th weeks are shown in Fig. 4A. Results of the micro-CT measurements of bone heights, widths, and volume are shown in Fig. 4B. Although middle graft height maintained throughout all time-points, the actual buccal and palatal bone heights and the bone volume on the grafted side were significantly less than the non-extraction side. However, the grafted side appreared to enhance bone width in comparison with the un-manipulated side. Representative histological sections of the maxillary first molar (M1) to third molar (M3) region of rats after M1 decoronation, roots extraction and Bio-Oss Collagen grafting were examined by hematoxylin & eosin (H&E) and Goldner's Trichrome staining at 4th, 8th, and 12th weeks (Fig. 4C). At 12th week after grafting, the defects grafted with Bio-Oss Collagen showed some bone regeneration in the defect site; however, the rate of new bone regeneration or mineralization of new bone were still not satisfactory at 12th week. Goldner's trichrome staining shows immature bone formation and soft tissue invasion were observed but no angiogenesis found.

## Discussion

**Figure 5:**
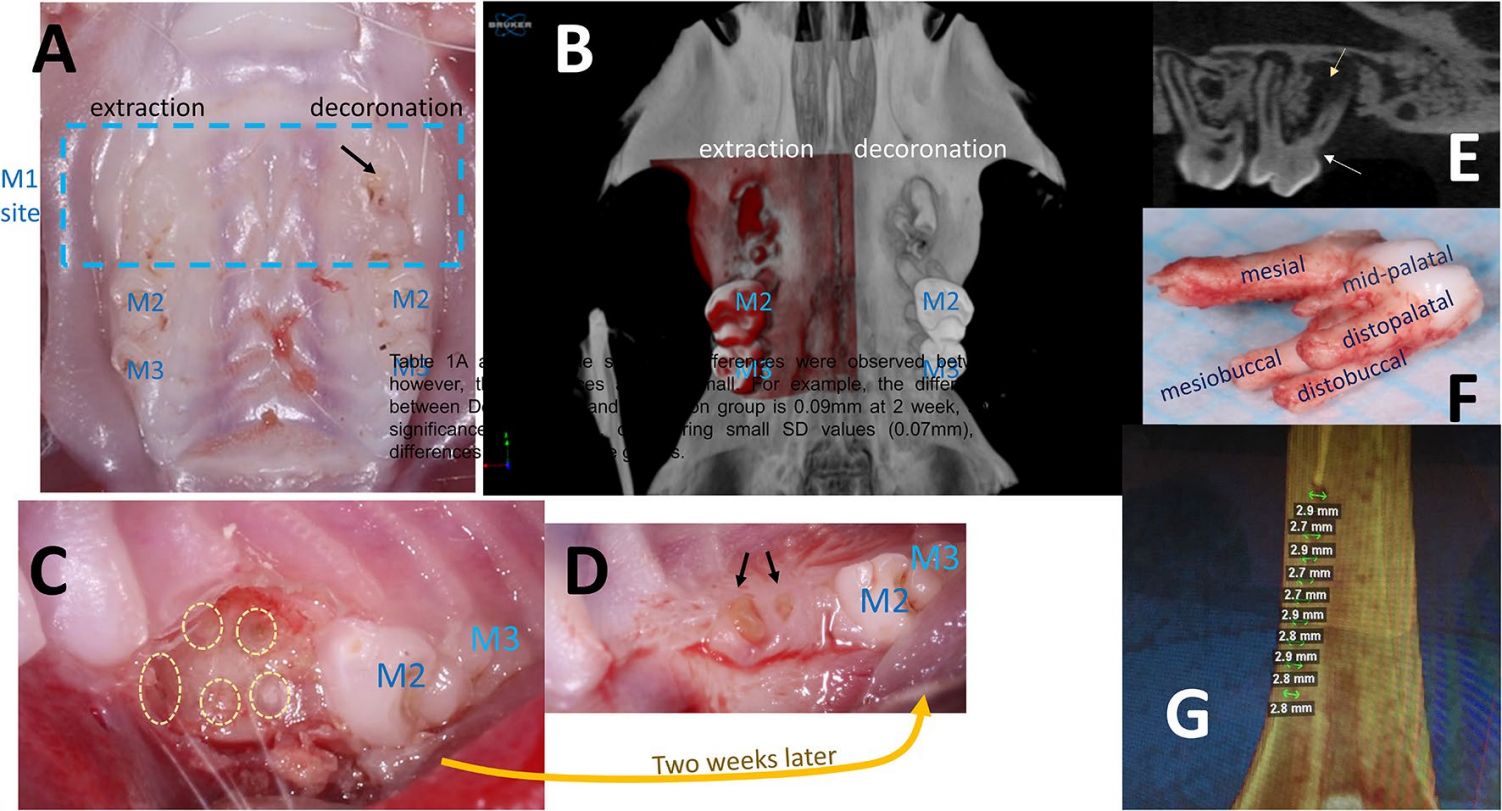
The effectiveness of preservation of alveolar ridge dimension associated with the decoronation procedure. (**A**) Representative image showing effectiveness of preservation of alveolar ridge dimension associated with the decoronation procedure at 6th week. (**B**) Superimposition of the mirror image of the decoronation side (red) in (**A**) to the extraction side. (**C**) Representative photo showing the maxillary first molar (M1) site just after decoronation. The bone and roots were left exposed for the secondary wound healing of the soft tissue. Yellow circles indicate the separated roots. (**D**) Representative photo showing 2 weeks after M1 decoronation. The soft tissue has grown well onto the decoronated M1 site. However, some roots have perforated through the soft tissue (black arrows). (**E**) Representative image of micro-CT section cut through the buccal aspect of M2 and M3 showing possible endodontic involvement (yellow arrow) caused by deep caries (white arrow) on the mesial root of M2. (**F**) Photo of an extracted M1 showing the slender mid-palatal root and the divergent mesial root. (**G**) In vitro testing of burr hole defects created on sawbone using diameter 2.7 mm tungsten carbide burr.

The root submergence technique was first introduced as an expedient and inexpensive way of preserving dimensions of the alveolar ridge and the surrounding tissues^21^ for the support of fixed partial denture in the esthetic zone^22^. In children and adolescents, decoronation is one the best treatment options for ankylosed tooth, this decoronation technique preserves the alveolar width and increases the vertical bone height of the alveolar ridge in growing individuals^23^. In this present study, we proposed using the decoronation or root submergence technique as a resort to preserve alveolar soft and hard tissue dimensions. Our results showed that alveolar bone height, crestal width, and 3D bone contour volume were considerably larger on the decoronation side (Fig. 5A,B), indicating a bigger housing which may potentially accommodate more grafting materials in future research.

Wang and Boyapati have described the PASS principles for predictable bone regeneration, namely, primary wound closure, angiogenesis, space maintenance/creation, and stability of wound^24^. Primary wound closure has a significant positive effect on the preservation of horizontal dimensions of alveolar ridge^25^. In the case of ridge preservation, soft tissues are often insufficient for achieving primary wound closure after tooth extraction especially in the molar region. When a large amount of augmentation material is required, the increased graft volume could cause tension in the soft tissue that may have resulted in soft tissue dehiscence. Exposure of graft increases the risk of infection and graft shrinkage because of compromised vascularization. Therefore, a mucoperiosteal flap is often created and mobilized via a periosteal release incision. This, however, may reduce the vascularity of the flap. In our study, we have decoronated rat maxillary first molar to the furcation level to separate the roots and allow the soft tissue to grow by secondary healing for at least 2 weeks (Fig. 5C,D). This decoronation technique has preserved the soft tissue height, thickness, and envelope to allow tension-free primary closure for subsequent grafting procedures. Rat maxillary molar roots may be extracted atraumatically and large bone defect may be created later for bone grafting.

Rat models have been used for experimental periodontal research^19,26^. As most rats are unsusceptible to develop natural periodontitis, periodontal disease has been induced by placing ligature around the cervical region of the molars to increase biofilm accumulation^27^. In alternative models, rats were inoculated with various periodontal pathogens or bacterial lipopolysaccharide^19,26^. However, these models were mainly used for microbiological, immunological, or pathogenetic studies. Rat models validating new regenerative therapies for damaged periodontal tissues usually involve surgically created bone defects^12,19,28^. However, both models were not relevant to natural periodontal disease and the defects were not infected. In our study, the decoronation process at M1 promoted periodontal breakdown especially at M2 mesial side. As evidenced from histologic and micro-CT findings, changes in the decoronation group included an intense infiltration of inflammatory cells and alveolar bone resorption characterized by an increase in the distance from CEJ to marginal bone starting from the 6th week. Possible explanation is that, although we have decoronated M1 to the level of furcation and the soft tissue has grown well onto the occlusal surface, some roots perforated through the soft tissue due to the continuous occlusal eruption of rat roots. The proximity of the exposed distal roots of M1 with the mesial roots of the M2 has promoted the development of periodontitis especially at the mesial side of M2. The severity of periodontal bone loss increased as the experimental period increased. However, deep caries on the mesial root of M2 in one rat might have resulted in endodontic-and-periodontal-combined lesion (Fig. 5E). Therefore, to use this as a periodontal (M2) alveolar bone defect (M1) model, care should be taken that the chance of pulp involvement complicating the situation could increase if the residual roots were left unextracted for a long time. Collectively with histological results, we would recommend 6th week after decoronation as the time-point to start subsequent periodontal alveolar bone defect research. Since the molar roots are separated during decoronation process, complete and atraumatic removal the five roots may be easily performed later; otherwise, as rat upper first molars have thin and diverged roots (Fig. 5F), the root fragments were frequently left behind during extraction of the rat tooth^12^.

Theoretically, the term ‘critical size defect’ is obscure when applying to alveolar ridge, because the presence and maintenance of the alveolar bone is ‘tooth-dependent’, which means that following tooth extraction alveolar bone will slowly resorb down to the body of the jawbone and will not heal spontaneously. However, if periodontally healthy adjacent teeth border the extraction site and the edentulous space is small, less bone resorption may be expected. Therefore, we will not stick to the term of ‘critical size’; instead, we recommend critical alveolar defect dimensions to accommodate maximum amount of grafting material if standardized size defect is preferred. The critical size of a bone defect may be associated with the species and age of animal^9,10^. When considering the entire life span together^29^, rats have more accelerated childhood in comparison with human and become sexually mature at about 6 weeks of age. The differences in the maturity of the selected animals may manifest the variations in their anatomy and developmental status, which should be taken into consideration while analyzing the results^13–16^. In this study, we have performed decoronation/extraction in 8-week-old SD male rats (equivalent to young adult) and provided the dimensions of the M1 site at 10th, 14th, 18th, and 22nd week (2, 6, 10, 14 weeks after decoronation, respectively) to estimate the critical dimensions for an alveolar defect to contain maximum amount of grafting material. In the middle of decoronated M1 sites, the most critical measurement was the vertical bone height, which ranged from 2.2 ± 0.3 mm to 2.3 ± 0.2 mm while the alveolar crestal width ranged from 2.8 ± 0.1 to 3.2 ± 0.3 mm. In our pilot study, we have additionally measured burr hole defects created on sawbones using diameter 2.7 mm tungsten carbide burr (ISO No. 027) and found the average defect diameter is 3.0 ± 0.1 mm and depth is 2.8 ± 0.1 mm (Fig. 5G). Considering that the middle alveolar crestal width, middle bone height, and middle soft tissue height of the decoronated M1 measured 3.2 ± 0.3 mm, 2.2 ± 0.2 mm, and 3.2 ± 0.2 mm, respectively at 2nd week; we suggest using burr size no larger than 2.7 mm when creating the defect in the middle of the M1 site to the full depth of the burr using soft tissue surface as a reference.

Eighteen additional 8-week-old SD rats were used to demonstrate the standardized size alveolar defect model using 2.7 mm diameter burr. The defects were created 2 weeks after decoronation, grafted with Bio-Oss Collagen, and followed up for another 4, 8, or 12 weeks. Bio-Oss, a deproteinized bovine bone mineral, is one of the most widely used biomaterial in ARP procedures. As Bio-Oss degrades slowly and may induce fibrous encapsulation with healing^30^, Bio-Oss Collagen, a composite of 90% Bio-Oss granules embedded in 10% porcine-derived collagen type I, has been introduced. Results from animal studies show that in comparison with non-grafted socket, the dimension of the alveolar process and the buccal profile of the ridge are better preserved with Bio-Oss Collagen grafting^31,32^. However, the slow resorption of the material may in fact delay healing^33,34^. Longitudinal follow-up shows that, Bio-Oss Collagen has the potential to limit but not entirely avoid the post-operative contour shrinkage^32^. Supplementary use of free gingival graft to seal the Bio-Oss Collagen-grafted socket seems to have no beneficial effect with respect to the contour alteration of the ridge^32^. Kim et al. have developed a chronic infection model by applying *Porphyromonas gingivalis* into the root canal and around the periodontium for four months. The infected mesial root sockets were used to evaluate Bio-Oss Collagen grafting with or without a collagen membrane^1^. This infection model is valuable in reproducing real clinical situations. However, the experimental procedures are complicated, the defect size is not standardized, and since the defect is created at only one root of the two-rooted premolar, defect size is still considered small relative to the jaw size of the dog. Clinical human trials using Bio-Oss Collagen to preserve alveolar ridge in intact sockets^35–38^ have concluded a positive role of Bio-Oss Collagen for ridge preservation. However, none of them have evaluated Bio-Oss Collagen grafting alone without collagen membrane or matrix. The likely benefit from barrier membrane or collagen matrix is overlooked. Thus, the clinical treatment effects from solely Bio-Oss Collagen grafting are still inconclusive. Our study showed that Bio-Oss Collagen alone could not fully preserve buccal or palatal bone height but should be beneficial in preserving ridge width in the extremely large alveolar defect rat model. It is also worth noticing that, by the end of observation period (12 weeks after grafting), grafting material was not completely resorbed and the graft height maintained.

## Conclusion

Collectively, we have demonstrated preservation of hard and soft tissue alveolar ridge dimension with the decoronation technique to allow accommodation of more grafting materials in subsequent research. If standardized critical dimension defect is preferred, we suggest extracting M1 roots 2 weeks after decoronation, and creating defect in the middle of M1 site with burr size no larger than 2.7 mm diameter to its full depth using soft tissue surface as reference. If infected alveolar bone defect at M1 with periodontally-involved M2 model is preferred, we suggest we suggest extracting M1 roots 6 weeks after decoronation to allow inflammation to occur. Additional rats had large standardized size alveolar defects grafted with Bio-Oss Collagen to confirm the protocol.

## Data Availability

The datasets used and/or analyzed in the current study are available from the corresponding author on reasonable request.
